# TERT RNAscope analysis of sub-centimetric papillary thyroid carcinomas and synchronous lymph node metastases

**DOI:** 10.1186/s13044-024-00195-7

**Published:** 2024-04-15

**Authors:** Marie-Lisa Eich, Wiebke Jeske, Uschi Zenz, Costanza Chiapponi, Christina Alidousty, Sabine Merkelbach-Bruse, Reinhard Büttner, Anne M. Schultheis

**Affiliations:** 1 Institute of Pathology, University Hospital Cologne and Medical Faculty, Cologne, Germany; 2 Institute of Pathology, Charité - Universitätsmedizin Berlin, corporate member of Freie Universität Berlin and Humboldt-Universität zu Berlin, Berlin, Germany; 3 Department of General, Visceral, Cancer and Transplant Surgery, University Hospital Cologne, Cologne, Germany

**Keywords:** *TERT*, Sub-centimetric papillary thyroid carcinoma, RNAscope®

## Abstract

**Background:**

Sub-centrimetric papillary thyroid carcinomas usually have a good prognosis with a cancer specific survival of > 99%, however in up to 65% of patients, lymph node metastases can be observed. Molecular alterations in *BRAF, TERT* and *TP53* are associated with worse clinicopathological outcome in patients with papillary thyroid carcinoma.

**Material and methods:**

Twenty-two cases of papillary thyroid carcinomas measuring ≤ 1 cm with synchronous lymph node metastases were examined regarding morphological patterns and immunohistochemical status of p53, Ki-67, and BRAF V600E status. *TERT* RNA expression in lymph node metastases were evaluated by RNAScope®.

**Results:**

Morphological patterns were heterogeneous in both primary tumors and lymph node metastases. Proliferation indices measured by Ki-67 were low. Both primary and lymph node metastases were wild type for p53 by immunohistochemical analysis. No lymph node metastasis showed *TERT* expression by RNAScope®.

**Conclusions:**

Our data indicate that *TERT* expression is not involved in the development early lymph node metastasis in patients with sub-centimetric PTC.

**Supplementary Information:**

The online version contains supplementary material available at 10.1186/s13044-024-00195-7.

## Introduction

Papillary thyroid cancer is the most common endocrine malignancy in both adults and children [1]. Several clinicopathological factors have been described to be associated with a more aggressive behavior, such as patient’s age, tumor size, extrathyroidal extension, lymph node metastasis, margin status, and subtype [2, 3]. However, the outcome in patients presenting with papillary thyroid carcinoma is highly heterogeneous.

With the advent of next-generation sequencing studies, the genomic background of the disease was unraveled. In papillary thyroid carcinomas, a high rate of activating somatic alterations in genes that encode effectors of the mitogen-activated protein kinase (MAPK) signaling pathway have been identified, including point mutations in the *BRAF* and *RAS* genes, the *TERT* promotor region, as well as fusions involving the *RET, ROS1*, *NTRK1* and *NTRK3* tyrosine kinases. *BRAF* mutations occur in approximately 55% of all papillary thyroid carcinomas, and specifically the *BRAF* V600E mutation is the most common genomic alteration. Meta-analyses have shown that patients with a *BRAF* V600E mutation, especially in combination with a *TERT* promoter mutation, have a higher risk of unfavorable clinicopathological parameters such as extrathyroidal tumor spread, lymph node metastasis, and a higher TNM stage [4–7]. Also, alterations in *TERT* and *TP53* can trigger progression from well-differentiated papillary thyroid carcinoma to more aggressive poorly differentiated thyroid carcinoma and anaplastic thyroid carcinoma [8, 9]. Genomic mechanisms of *TERT* upregulation and reactivation include *TERT* promotor mutations as the most common one. However, there are also other mechanisms that lead to overexpression of *TERT* mRNA. Recently, an assay for RNA in situ hybridization RNAScope® has been developed with the advantage of capturing all RNA aberrations leading to increase RNA expression.

Sub-centrimetric papillary thyroid carcinomas show in general a better clinical behavior with a cancer specific survival of > 99% [10]. They were classified as a distinct subtype of papillary thyroid carcinomas, namely papillary thyroid microcarcinoma, in the previous WHO classification. However, depending on the study, lymph node metastases have been described in up to 65% of patients [11, 12].

Therefore, our aim was to examine the role of *TERT* expression level alterations using RNAscope®, and histological patterns in a cohort of papillary thyroid carcinoma patients with a tumor size ≤ 1 cm harboring synchronous lymph node metastasis.

## Methods

### Patient cohort

The study was conducted in accordance to local ethical guidelines. Patient consent has been obtained from each patient after full explanation of the purpose and nature of the all procedures used, and approval by the ethical board was granted (number 21-1025_1, institutional review board of the University Hospital of Cologne).

Twenty-two specimens of papillary thyroid carcinoma with a tumor size ≤ 1 cm (traditionally classified as papillary thyroid microcarcinoma) and corresponding lymph node metastases diagnosed between 2012 and 2022 were retrieved from the surgical pathological archives. No prophylactic lymphadenectomy is performed in case of tumors ≤ 1 cm at our institution, according to the current German Guidelines. Accordingly, all patients had either preoperative sonographic or biopsy diagnosis of suspicious lymph nodes. In four patients papillary carcinoma lymph node metastases were detected on lymph node dissection for head and neck squamous cell carcinoma, followed by subsequent thyroidectomy. Lymph node metastasis removed later than 6 months after diagnosis were considered metachronous and were not included in the present study, as well as all with concomitant carcinoma > 1 cm.

### Histopathological evaluation

Whole slide images of thyroid resection specimens and lymph node dissection samples were evaluated by two experienced pathologists (AMS, MLE). For establishment of diagnosis of lymph node metastasis of papillary thyroid carcinoma TTF1 and PAX8 immunohistochemistry was performed in routine diagnostic workflow. Positivity of TTF1 and PAX8, indicating thyroid origin along with morphologic features, lead to the diagnosis of lymph node metastasis from papillary thyroid carcinoma. The concomitant lymph node metastasis from head and neck squamous cell carcinoma were negative for these two markers.

Both primary tumors and lymph node metastases were examined with regard to presence of stromal desmoplasia, histologic variant of papillary thyroid carcinoma (infiltrative follicular, classic, and others), presence of inflammation, psammoma bodies, and extracapsular extension. Lymphovascular invasion in primary tumors was also examined, as well as the presence of underlying Hashimoto’s thyroiditis.

### Immunohistochemical assessment

Whole slide sections of primary tumor and the largest lymph node metastasis were used to perform immunohistochemistry for p53 (clone: DO-7. 1:1800, Dako / CE), Ki-67 (clone: SP6. 1:100. Cellmarque / CE) and BRAF V600E (clone: VE1, ready to use, Roche / CE) following the manufacturer’s protocol. Detection of immunolabeling was performed using anti-mouse or anti-rabbit horseradish peroxidase–conjugated secondary antibodies and developed using 3,3′-diaminobenzidine.

For BRAF V600E diffuse cytoplasmic staining of tumor cells was considered positive and used as a surrogate marker for an underlying mutation. P53 was considered abnormal, if a strong nuclear expression in a majority of nuclei was present, a strong cytoplasmic staining or the absence of staining. A varying staining pattern across tumor nuclei was considered wild type staining pattern.

For evaluation of proliferation index via Ki-67, whole slide images were digitized with a Hamamatsu S360 scanner. Representative tumor areas were annotated in QuPath 0.3.2. Positive cells were detected running the built-in positive cell detection command using following parameters: detection image optical density sum, requested pixel size 1 µm; nucleus parameters: background radius 8 µm, median filter radius 0 µm, sigma 2 µm, minimum area 12 µm2, maximum area 400 µm2; intensity parameters: threshold 0.1, max background intensity 2; and cell parameters: cell expansion 5 µm, cell nucleus included.

### *TERT* RNAscope®

The largest lymph node metastasis of each case was selected for analysis. RNAscope® detection for *TERT* was performed manually using the Hs-TERT-01 probe. Staining was done a per manufacturer’s protocol (ACDBio). Appropriate accompanying positive and negative controls (universal negative control dapB probe) were used to evaluate the staining procedure. After pre-treatment, probe hybridization and detection, the slides were counterstained with 50% hematoxylin, dehydrated, and mounted.

Expression quantification was carried out as described by Momeni-Boroujeni et al. [13] TERT signals in 100 cells were counted in hotspot regions.

## Results

### Clinicopathological parameters

Table 1 summarizes the clinicopathological parameters of the 22 patients with sub-centimetric papillary thyroid carcinomas with simultaneous lymph node metastasis identified at our institution from 2012 to 2022. Age and gender for each case can be found in Supplementary Table 1, as well as location (central and lateral compartment) of lymph node metastases.

**Table 1:** Clinicopathological parameters in 22 patients with sub-centimetric papillary thyroid carcinoma and synchronous lymph node metastases

Variables	Values
*n*	22
Age, years	
Mean (± SD)	48 (14.5 ± SD)
Min, max	19, 80
Sex (%)	
Male	13 (59%)
Female	9 (41%)
pT stage (%)	
pT1a	22 (100%)
pN stage	
pN1a	3 (14%)
pN1b	19(86%)
Tumor size in cm	
Mean (± SD)	0.52 (± 0.27)
Lymph node metastasis size in cm	1.64
Mean (± SD)	± 1.15
Multifocality (%)	13 (59%)

Four patients (patient 4, patient 9, patient 13, and patient 17) showed simultaneous head and neck squamous cell carcinomas (patient 4: squamous cell carcinoma of tonsil; patient 9 squamous cell carcinoma of floor of the mouth, patient 13: squamous cell carcinoma of tonsil and patient 17: squamous cell carcinoma of larynx). Regarding outcome, data was available for 15 patients. One patient experienced disease recurrence in a cervical lymph node (see Supplementary Table 1).

### Comparison of morphological features between primary tumors and lymph node metastasis

The morphological features of primary tumors and their lymph node metastases are summarized in Supplementary Table 1. Sixteen primary tumors were of classic and 6 of infiltrative follicular subtype, other subtypes were not observed. In patients 1, 2, and 14, the subtype differed between primary tumors and lymph node metastases. In patient 1, the primary tumor was of classic subtype, whereas the lymph node metastases showed follicular morphology. In patients 2 and 14, the primary tumor was composed of papillae and showed classic morphology, whereas the lymph node metastases were follicular. Lymphovascular invasion could not be detected morphologically in any of the tumors. Stromal dysplasia was present in 16 of primary tumors and 8 lymph node metastases. The size of primary tumors ranged from 0.3 to 10 mm and from 1.5 to 45 mm for the accompanying lymph node metastases. Extranodal growth was seen in four specimens. Fourteen primary tumors were BRAF V600E mutated by immunohistochemical evaluation. Of these, thirteen displayed classic morphology, and only one showed absence of stromal dysplasia. In thirteen of the corresponding lymph nodes, a mutational pattern was observed. In one case, no tissue was left for further evaluation (see Supplementary Table 1).

### Immunohistochemical expression patterns of p53 and Ki-67 in primary tumors and lymph node metastasis

**Fig. 1:**
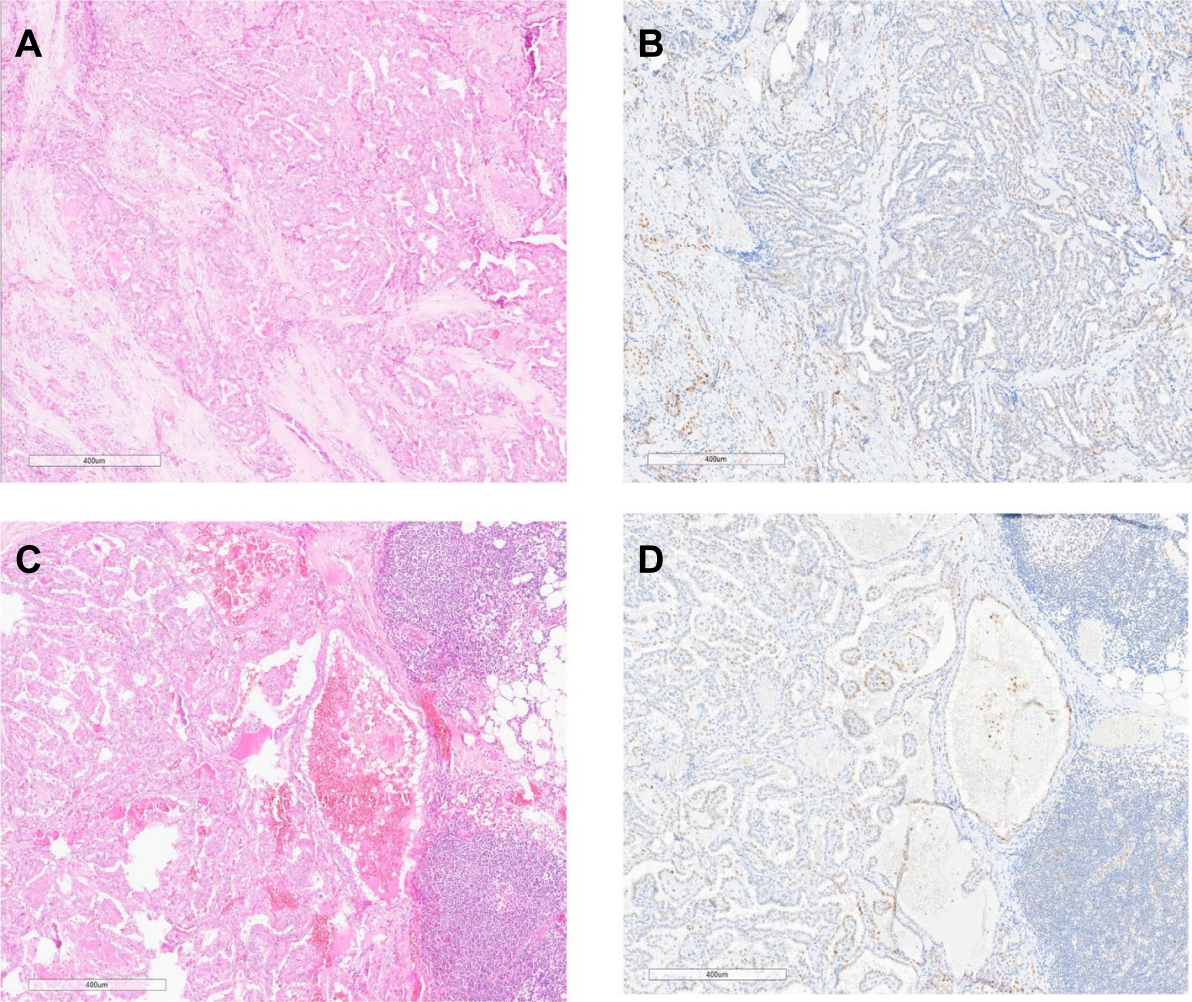
p53 expression pattern in sub-centimetric papillary thyroid carcinoma and accompanying lymph node metastases. HE- and p53 immunohistochemical staining in small papillary thyroid carcinoma (**A**, **B**) and lymph node metastases (**C**, **D**) in patient 13

**Fig. 2:**
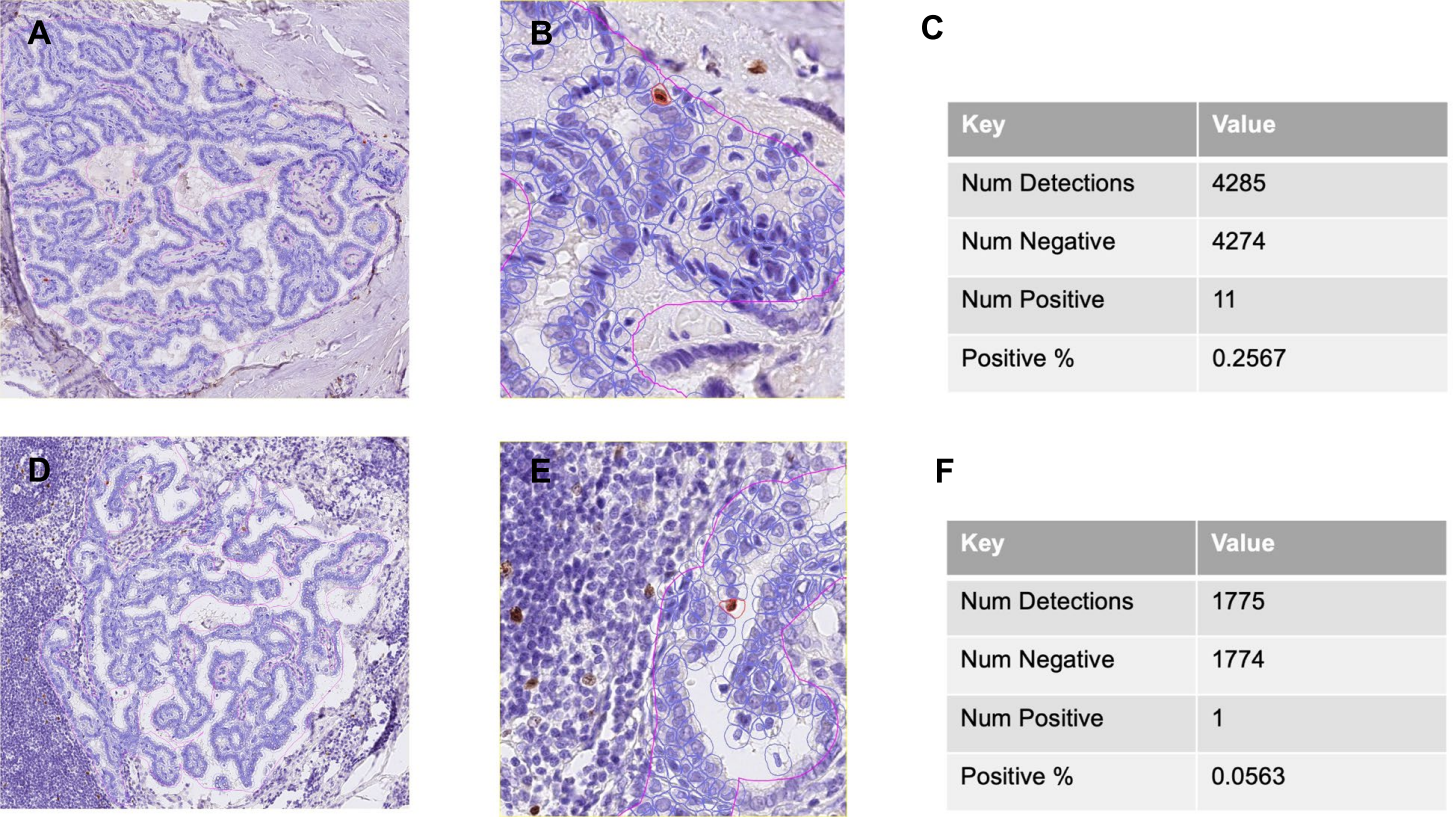
Ki-67 count in sub-centimetric papillary thyroid carcinoma and accompanying lymph node metastases. Ki-67 staining and detection pattern in papillary thyroid carcinoma (**A**, **B**, **C**) and lymph node metastases (**D**, **E**. **F**). B and E represent higher magnification images of A and D. Num Detections: number of detected cells. Num Positive: number of positive cells. Num negative: number of negative. % positive: % of Ki-67 positive cells

Tissue for immunohistochemical analysis of p53 and Ki-67 was available in 18 of primary tumors and all lymph node metastases. The P53 staining pattern was considered wild type in all specimens. Proliferation rates measured by Ki-67 ranged from 0.05 to 2.69% in primary and from 0.22 to 2.56% in lymph node metastases (see Table 2). Representative tumors are shown in Figs. 1 and 2.

**Table 2:** p53 and Ki-67 expression patterns in primary tumors and lymph node metastases in patients with sub-centimetric papillary thyroid carcinomas

**Case**	Thyroid		Lymph node metastases	
	**P53-IHC**	**Ki-67 (%)**	**P53-IHC**	**Ki-67 (%)**
PTMC1	NA	NA	WT	2.16
PTMC2	NA	NA	WT	1.73
PTMC3	NA	NA	WT	2.15
PTMC4	WT	0.6	WT	0.54
PTMC5	WT	0.86	WT	0.42
PTMC6	WT	0.23	WT	0.78
PTMC7	WT	0.68	WT	0.24
PTMC8	WT	0.73	WT	2.17
PTMC9	WT	0.75	WT	2.46
PTMC10	WT	2.69	WT	0.3
PTMC11	WT	0.55	WT	1.37
PTMC12	WT	1.63	WT	1.76
PTMC13	WT	0.06	WT	0.66
PTMC14	WT	0.4	WT	0.33
PTMC15	WT	0.37	WT	0.37
PTMC16	WT	1.4	WT	6.76
PTMC17	WT	0.26	WT	0.06
PTMC18	WT	0.04	WT	2.43
PTMC19	WT	0.57	WT	0.54
PTMC20	WT	0.05	WT	1.32
PTMC21	NA	NA	WT	0.22
PTMC22	WT	0.55	WT	0.93

### *TERT* status by RNAscope® in lymph node metastases

**Fig. 3:**
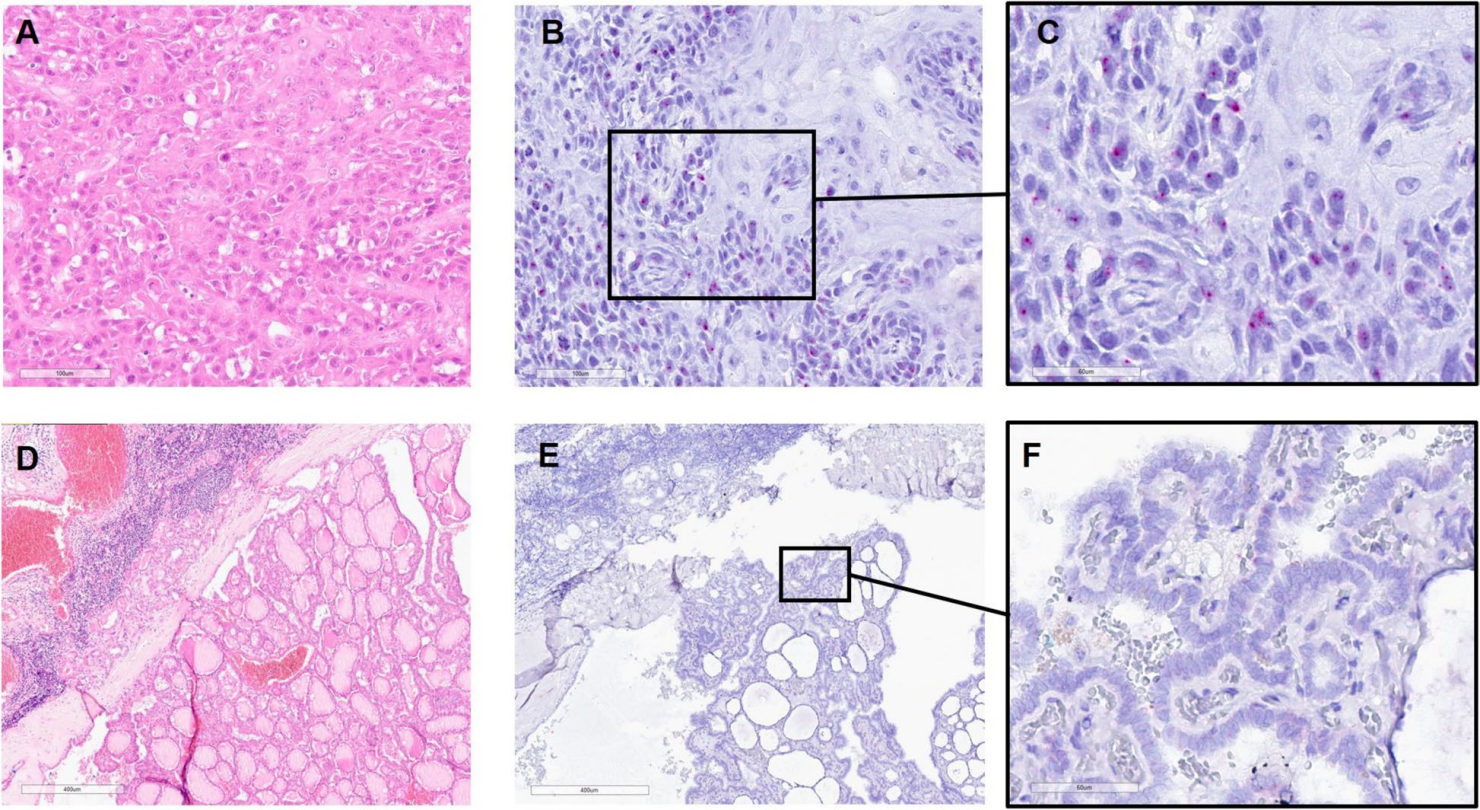
TERT RNAScope® in lymph node metastases from sub-centimetric papillary thyroid carcinoma. *TERT* RNA expression in lymph node metastases from squamous cell carcinoma (HE: A, TERT RNA Scope®: B, C) and papillary thyroid carcinoma (HE: D, TERT RNA Scope®: E, F). Scale bar 100 µm (**A**, **B**), 400 µm (**D**, **E**), 60 µm (**C**, **F**)

None of the 22 specimens showed *TERT* expression using RNAscope. In one of the tumors with accompanying lymph node metastases from squamous cell carcinoma, *TERT* expression was observed in squamous cell carcinoma in the basal portion of the tumor while being absent in superficial cell layers, as well as in the metastasis of papillary thyroid carcinomas (Fig. 3).

## Discussion

Although erased as a papillary thyroid carcinoma subtype in the updated 5th WHO classification system, papillary carcinomas measuring 1 cm or less were considered a subclass of papillary thyroid carcinomas and were included from 2004 until 2022 in the WHO classification of thyroid tumors under the term “microcarcinomas”. They are frequently incidental findings and traditionally show good clinical behavior. However, studies have observed lymph node metastasis in up to up to 65% of patients [11]. A more recent observational study including 293 patients with papillary thyroid microcarcinoma from 2003 to 2019 detected lymph node metastasis in 13.7% of patients, which were clinically unexpected in 9.8% and suspected in 3.8%. The authors found patient’s age < 45, tumor size ≥ 0.6 cm, the tall cell variant of papillary thyroid carcinoma, extrathyroidal extension and angioinvasion to be risk factors for lymph node metastasis on multivariate analysis [12]. In the study of 551 patients with papillary thyroid microcarcinomas by So et al., male gender, tumor multifocality, and extrathyroidal extension were predictive factors for lymph node metastasis on multivariate analysis. Their incidence on lymph node positivity was 37% [14]. Similarly, Gu et al. detected lymph node metastasis in 29.5% of their 268 studied patients with papillary thyroid microcarcinomas and found male sex, younger patient’s age, and tumor diameter on ultrasound > 0.5 cm to be risk factors for nodal involvement [15].

In our study, the mean tumor size was 0.52 cm. Additionally, the presence of other histological features like stromal desmoplasia, encapsulation, and multifocality were inconsistent in our group of patients with papillary thyroid carcinomas measuring ≤ 1 cm and harboring lymph node metastasis. Furthermore, *BRAF* V600E mutations have been shown to be a poor prognostic factor with regard to persistence of disease, recurrence, and overall survival [6, 7, 16]. In particular, the presence of coexisting *BRAF* V600E and *TERT* promoter mutations is associated with worse clinical outcome [17–21]. Studies on small papillary carcinomas (papillary thyroid microcarcinomas) found hotspot *TERT* promotor mutations in 0 to 8.7% of tumors [22–24]. Other studies have shown that not only *TERT* promotor mutations with the common hotspots C228T and C250T are linked to adverse outcome, but also aberrant *TERT* promoter methylation patterns, gains of the *TERT* gene locus at chromosome 5p15.33, and TERT mRNA overexpression [25–27]. When looking at TERT mRNA levels Pestana et al. found in their study of 244 thyroid samples malignant tumors to have higher TERT mRNA expression compared to benign lesions. The highest mRNA levels were found in more aggressive histotypes like poorly differentiated thyroid carcinoma, but also papillary thyroid carcinoma. These are known to harbor the highest frequencies of TERT alterations among thyroid lesions. Furthermore, mRNA expression was also found in the absence of *TERT* promotor mutations [28]. Generally, sequencing studies are time consuming and not always feasible, due to small tumor size and limited material. So far, there is no reliable IHC assay for TERT expression on the market [29]. Recently an assay for RNA in situ hybridization RNAscope® has been developed with the advantage of capturing all RNA aberrations leading to increase RNA expression. Examining the correlation between RNA-sequencing and the RNAscope® assay in 48 different tissue types gave promising results, while the in-situ hybridization method has the advantage of visualizing the expression pattern on a cellular level, making it possible to identify heterogenous *TERT* expression in different cell types [13]. All examined lymph nodes from our cohort of papillary thyroid “microcarcinomas” were negative for *TERT* using RNAscope®. This goes into a similar direction as the study by Lee et al. who examined *TERT* promotor mutation status in combination with *BRAF* V600E mutations in a cohort of 504 consecutive patients with sub-centimetric papillary thyroid carcinomas measuring. They found a co-occurrence of *TERT* and *BRAF* alterations in 16 (3.2%) patients but no association with lymph node metastasis [30]. Another study looking at 16 patients with small papillary carcinomas and their lymph node metastases found *BRAF* V600E mutation in 75% of tumors, but a low mutational burden in both lymph node metastases and primary tumors. No* TP53* mutations were observed in this study; *TERT* promotor mutations were not examined [31]. Also, we did not observe an abnormal p53 expression by immunohistochemistry as a surrogate parameter for an underlying *TP53* mutation. Perera et al. also identified *TP53* mutations in only 1% of their cohort of small papillary thyroid carcinomas with neck lymph node metastases and mutations were restricted to pN1b tumors [32].

In summary, we have examined 22 papillary thyroid carcinomas, measuring ≤ 1 cm with simultaneous lymph node metastasis, in regard to their *TERT* mRNA expression status, BRAF V600E and p53 status. We did not observe any expression of *TERT* mRNA in the lymph node metastasis, as well as no abnormal p53 expression, both in primary tumors, as well as the accompanying lymph node metastases. With respect to morphology, 16 lesions showed a classic pattern, while 6 belong to the follicular subtype. Stromal dysplasia, encapsulation and lymphoid infiltrate varied across primary tumors.

## Conclusion

Taken together, our data indicate that *TERT* expression level alterations are not involved in the development of early lymph node metastasis in patients with sub-centimetric papillary thyroid carcinomas.

As our sample size is relatively small and *TERT* promotor mutations were not evaluated in this study, subsequent prospective studies confirming our findings are needed and are in preparation. In particular, studies are warranted exploring novel molecular marker and or morphological factors for the prediction of early lymph node metastasis to better risk stratify patients with small tumors.

### Supplementary Information

**Supplementary Material 1.**

## Data Availability

All data can be provided upon request.
